# Pre-procedural C-reactive protein levels and carotid or intracranial artery restenosis: A systematic review and meta-analysis

**DOI:** 10.1016/j.athplu.2026.01.006

**Published:** 2026-01-31

**Authors:** Krishi Jain, Hermann Pasha, J.J. Coughlan, Daniel O'Callaghan, Niall Connolly, Roisin Colleran, Osama Soliman, Robert A. Byrne, Himanshu Rai

**Affiliations:** a Cardiovascular Research Institute (CVRI) Dublin and Department of Cardiology, Mater Private Network, Dublin, Ireland; b School of Medicine, RCSI University of Medicine and Health Sciences, Dublin, Ireland; c School of Pharmacy and Biomolecular Sciences, RCSI University of Medicine and Health Sciences, Dublin, Ireland; d School of Anatomy and Regenerative Medicine, RCSI University of Medicine and Health Sciences, Dublin, Ireland

**Keywords:** C-reactive protein, Carotid artery disease, Intracranial stenosis, Restenosis, Meta-analysis

## Abstract

**Introduction:**

Restenosis of the carotid or intracranial (IC) arteries, shown to be associated with increased risk of ischemic stroke, represents an unresolved clinical issue. Residual local and systemic inflammation at the time of the index revascularization, of which C-reactive protein (CRP) is a marker of, has been associated with coronary restenosis. Its association with carotid or IC restenosis post revascularization, however, remains uncertain. We therefore conducted a systematic review and a study-level meta-analysis investigating the association of pre-procedural CRP levels and the subsequent incidence of carotid and IC restenosis.

**Methods:**

Online databases of PubMed, EMBASE, MEDLINE, Scopus, and Web of Science were systematically searched for articles published until August 31st, 2025. Pooled effect sizes were obtained from study-level standard mean differences (SMD) and their 95% confidence intervals (CI), employing a Z-test, with random effects for analysis.

**Results:**

Out of 175 unique articles screened, 12 case-control studies, with a total sample of 2040 (325 restenosis cases/1715 no-restenosis controls), were included for quantitative synthesis. The pooled results demonstrated that pre-procedural CRP levels were associated with carotid or IC restenosis, with significantly higher pre-procedural CRP levels amongst the restenosis group in comparison to the no-restenosis group (SMD = 0.50, 95% CI = 0.11–0.89, p = 0.01). No apparent publication bias was detected either visually by Begg's funnel plot or Egger's test (p = 0.51). Leave-one-out sensitivity analysis supported the robustness of the results.

**Conclusions:**

This meta-analysis suggests a significant association between pre-procedural CRP levels and the subsequent incidence of carotid and intracranial artery restenosis.

## Highlights

• Restenosis of the carotid or intracranial (IC) arteries is associated with stroke.
• Elevated C-reactive protein (CRP) levels suggest local and systemic inflammation.
• CRPs association with carotid or IC restenosis was investigated via a meta-analysis.
• We demonstrate a significant association, suggesting elevated CRP as a risk factor.

## 1 Introduction

Atherosclerotic stenosis of the carotid and intracranial (IC) arteries are associated with an increased risk of stroke [[1], [2]], [[1], [2]] which, according to the World Stroke Organization is the second leading cause of death and disability amongst non-communicable diseases [3].

For patients undergoing intervention for carotid or IC stenoses, investigated treatments have included endarterectomy, stenting, plain old balloon angioplasty and drug coated balloon angioplasty. Restenosis, which is the re-narrowing of revascularized carotid and IC lesions remains a clinical concern after carotid and IC revascularization [4,5]. The International Carotid Stenting Study, which was the largest multicenter, parallel group randomized trial to date, reported that the cumulative 5-year incidence of moderate (≥50%) and severe restenosis or occlusion (≥70%) to be 40.7% and 10.6% following carotid endarterectomy [4]. On the other hand, moderate or severe restenosis or occlusion rates at 5-year following carotid stenting were found to be slightly lower, but still clinically substantial (29.6 ad 8.5% respectively) [4]. Notably, both of aforementioned percentage based restenosis cut-offs were associated with increased risk of subsequent ipsilateral strokes [4]. Similarly, the pooled restenosis rate after IC stenting has been reported to be 14.8%, in a meta-analysis of 51 published studies. Patients returning with carotid and IC restenosis often require re-intervention and have poor prognosis, in terms of an increased risk of recurrent strokes or transient ischemic attacks (TIAs) [4,6]. This highlights that restenosis in carotid and IC lesions post intervention is clinically important and portends an increased risk of adverse outcomes [5].

Residual inflammation at the time of index revascularization has been associated with restenosis [7], and C-reactive protein (CRP) is a marker of inflammation. CRP is an acute phase reactant; a pentameric protein synthesized in the liver in response to inflammation [8]. It is a widely used marker of inflammation, easily estimable from peripheral blood, and has been independently associated with adverse cardiovascular events such as myocardial infarction, stroke and cardiovascular death [9]. Elevated pre-procedural CRP levels have been associated with an increased risk of clinical outcomes after coronary revascularization [[10], [11]], [[10], [11]] including coronary restenosis [12]. However the association between CRP and the incidence of carotid or IC restenosis has not been established.

Against this background, we conducted a study-level meta-analysis investigating the association of pre-procedural CRP levels with the incidence of carotid and IC restenosis.

## 2 Materials and methods

The present work strictly adhered to the PRISMA 2020 statement (Preferred Reporting Items for Systematic Reviews and Meta-Analyses, PRISMA - updated 2020 guidelines) [13].

### 2.1 Search strategy and study selection criteria

Online databases of National Library of Medicine (PubMed), EMBASE, MEDLINE-OVID, Scopus and Web of Science were systematically searched for relevant articles published online until August 31, 2025. Search strings were constructed for each of the databases using specific MESH headings, in combination with open text fields. Supplementary Table 1. lists the search strings used for each online database, along with the number of results retrieved. Common search terms used for building precise search strings were “C-reactive protein” OR “high sensitivity C-reactive protein” AND “angioplasty” OR “stent” OR “endovascular treatment” OR “transluminal treatment” OR “intervention” OR “bypass” OR “reconstruction” or “endarterectomy” AND “carotid artery disease” OR “carotid arter∗” OR “Intracranial arterial disease” OR “Intracranial arter∗” OR “intracranial” OR “carotid” AND “restenosis” OR “re-stenosis” OR “revasculari∗” OR “re-intervention” OR “repeat intervention” OR “disease progression” OR “recurrent stenosis” NOT “rat” OR “mouse” OR “mice” OR “rabbit” OR “Takayasu arteritis”. References sections of included papers and relevant published meta-analyses were also reviewed to identify additional articles. Our search was limited to only original articles, published in English language, involving human subjects. Study titles were assessed, followed by abstracts, and subsequently full texts for potential inclusion.

### 2.2 Inclusion and exclusion criteria

Qualifying studies had to have clearly defined restenosis cases and no-restenosis controls. The definition of restenosis was based either on visual estimation by operators or using offline detailed assessment, with ≥50% stenosis or individual study-based cut-offs for diameter stenosis inside the previously treated segment. Visual confirmation, or the absence of ≥50% stenosis or individual study-based cut-offs, defined our controls classified as having no-restenosis. The design of the included studies could have been case-control/cohort based. No restrictions were put either on: (i) procedure type (carotid/intracranial stenting or carotid endarterectomy, or (ii) the assay type (CRP or high-sensitivity CRP), or (ii) the duration from index procedure to follow-up restenosis assessment.

### 2.3 Data collection

Data from the included studies for quantitative synthesis was independently extracted by two assessors. Raw extracted data, as well as relevant clinical characteristics was collected on separate Microsoft excel spreadsheets, any discrepancy amongst the two were resolved by deliberations. Summary CRP values amongst the restenosis and no-restenosis groups, expressed as means and standard deviations (mean ± SD), were used for quantitative synthesis. Transformation of the data (if required) was later performed using standard methodologies [14,15].

### 2.4 Quality assessment

The Newcastle-Ottawa scale (NOS, https://www.ohri.ca/programs/clinical_epidemiology/oxford.asp) was used for quality assessment of the included studies. NOS is a star-based rating system, which considers selection of cases and controls, comparability between cases and controls, and exposure, where a good study will be rated between 5 and 9 stars.

### 2.5 Statistical analysis

All statistical analysis was performed using review Manager (RevMan) [Computer program]. Version 5.4.1 The Cochrane Collaboration, 2020 and GraphPad Prism for Windows Version 10.6.1.

#### 2.5.1 Summary effect measures

Study level standard mean differences (SMD) and their 95% confidence intervals (CIs) were pooled to obtain a pooled SMD and 95% CI by employing a Z test using the more conservative, random effect models (DerSimonian-Laird method) [16]. A resulting p value of <0.05 was taken to indicate statistical significance. On the contrary, fixed effects model (Mantel-Haenszel method) [17], normally used for study groups displaying low levels of heterogeneity, was not used for the presented analysis, anticipating high levels of inherent in-group heterogeneity.

#### 2.5.2 Heterogeneity assessment

A Q test was employed for heterogeneity assessment in the analysed study group, where a resulting Higgin's *I*^*2*^ statistics (*I*^*2*^) along with Cochran's Q statistics (P_Q_) were considered as heterogeneity indicators. Significant in-group heterogeneity was indicated by a P_Q_ of <0.01, whilst *I*^*2*^ values of 25%, 50% and 75% indicated low, moderate and high heterogeneity respectively [18].

#### 2.5.3 Publication bias assessment

Publication bias amongst the group of included studies was visually assessed using Begg's funnel plots, where the study level SMD was plotted against its standard error [19]. Additionally, Egger's test was employed, which provided statistical estimates for the same, with a p value < 0.05 taken to indicate statistical significance [20]. Funnel plot asymmetry, coupled with a significant Egger's p value, would suggest the possible existence of publication bias.

#### 2.5.4 Sensitivity analysis

Leave-one-out sensitivity analysis was performed by excluding one study after another and repeating the analysis each time, testing for any significant deviation from the originally obtained pooled results. No significant deviation after each repeated analyses was taken to indicate the robustness of the obtained results.

## 3 Results

**Fig. 1:**
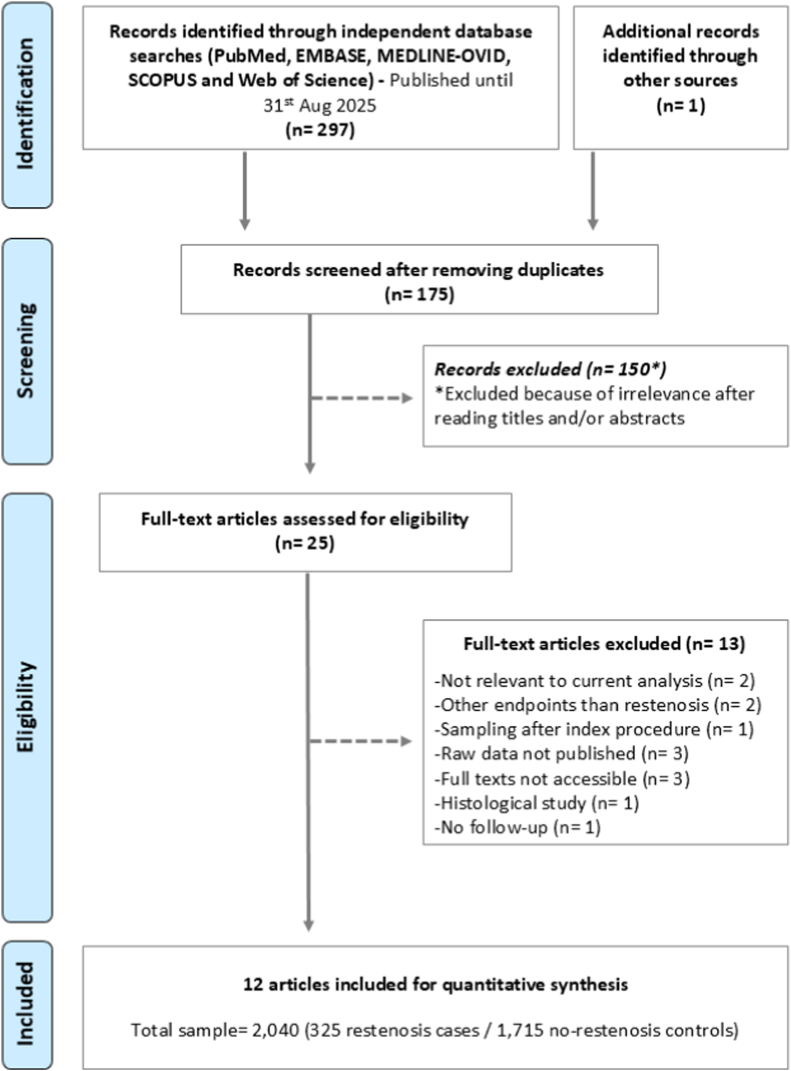
PRISMA study flow.

Out of a total of 298 retrieved records, 175 were identified to be unique and underwent title and abstract level screening. Out of these, 150 records were excluded because they were not relevant to the current analysis, leaving 25 articles to be assessed via full text screening. Of these, 13 articles were excluded for a variety of reasons, as detailed in Supplementary Table 2. This resulted in a total of 12 articles/studies, with a total sample of 2040 (325 restenosis cases and 1715 no-restenosis controls) included for quantitative synthesis [[21], [22], [23], [24], [25], [26], [27], [28], [29], [30], [31], [32]]. The PRISMA flow diagram is depicted in Fig. 1**.**

Table 1 summarizes the baseline characteristics of the included studies, including the type of index revascularization procedure, the CRP units in which the values were reported, sample sizes, restenosis definitions and its assessment methodology, the duration between the index procedure and follow-up restenosis assessment and NOS rating. All included studies were adjudged to be of good quality with NOS ratings ranging from 6 to 9 stars.

**Table 1:** List of included studies.

Author	Year	Procedure	Study design	CRP units (as reported)	Total Sample	Restenosis (n)/No-restenosis (n)	Restenosis definition (% stenosis)	Restenosis assessment methodology	Duration from index procedure to follow-up restenosis assessment	New-castle Ottawa scale rating
Schillinger et al. [21]	2003	Carotid artery stenting	Prospective cohort	mg/dl	108	15/93	>50%	Doppler ultrasound	6 months	7/9
Gupta et al. [22]	2010	Extracranial and intracranial stenting	Retrospective case-control	mg/dl	73 (57 extracranial, 16 intracranial)	9/64	>50%	Repeat angiography	>3 months	7/9
Wasser et al. [23]	2011	Carotid artery stenting	Prospective cohort	mg/L	210	12/198	≥70%	Doppler ultrasound	33.4 months	7/9
Xia et al. [24]	2012	Carotid artery stenting	Prospective cohort	mg/L	61	14/47	≥11%	Computed tomography angiography	12 months	7/9
Shi et al. [25]	2016	Carotid artery stenting	Retrospective case-control	mg/L	57	11/46	>50%	Cerebral angiography	6 months	6/9
Tanaskovic et al. [26]	2018	Eversion Carotid Endarterectomy	Prospective cohort	mg/L	285	26/259	>10%	Doppler ultrasound	24 months	7/9
Guo et al. [27]	2021	Intracranial stenting	Retrospective case-control	mg/L	97	24/73	≥50%	Digital subtraction angiography	Median 12.7 months	8/9
Haidegger et al. [28]	2021	Intracranial stenting	Prospective cohort	mg/L	115	38/77	≥50%	Transcranial Duplex sonography	5 years (1, 3, 6 months and then annually)	7/9
Yu et al. [29]	2021	Intracranial stenting	Retrospective case-control	mg/L	279	80/199	>50%	Digital subtraction angiography and computed tomography angiography	Mean 11 months, median 9 months	6/9
Liu et al. [30]	2023	Carotid artery stenting	Prospective cohort	mg/L	296	28/268	>50%	Doppler ultrasound	Mean 48 months (1, 6, 12 and yearly thereafter)	7/9
Chen et al. [31]	2025	Intracranial paclitaxel coated balloon treatment	Prospective cohort	mg/L	261	35/226	>50%	Digital subtraction angiography	6 months	7/9
Luo et al. [32]	2025	Extracranial and intracranial stenting	Retrospective case-control	mg/L	198 (Extracranial stenting + intracranial stenting - numbers not specified)	33/165	>50%	Digital subtraction angiography and computed tomography (neck)	6–12 months	8/9

**Fig. 2:**
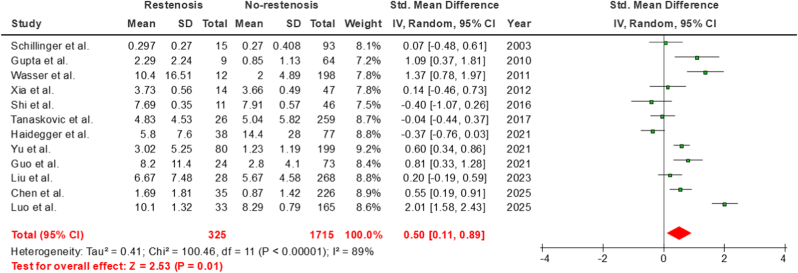
Main results.

**Fig. 3:**
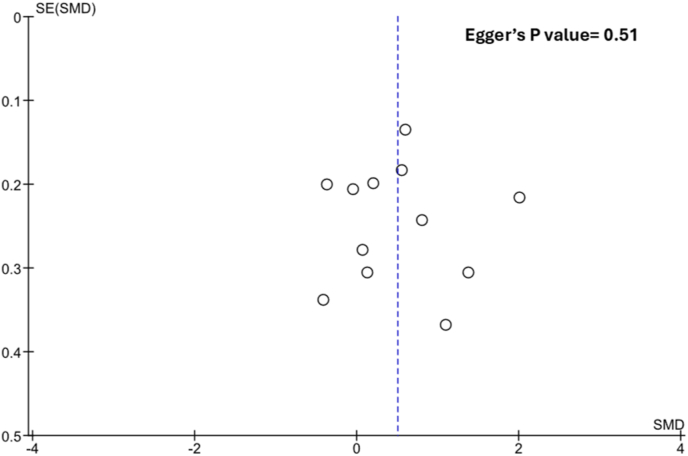
Publication bias assessment using Begg's funnel plot and Egger's test Abbreviations: SE, standard error; SMD, standard mean difference.

**Fig. 4:**
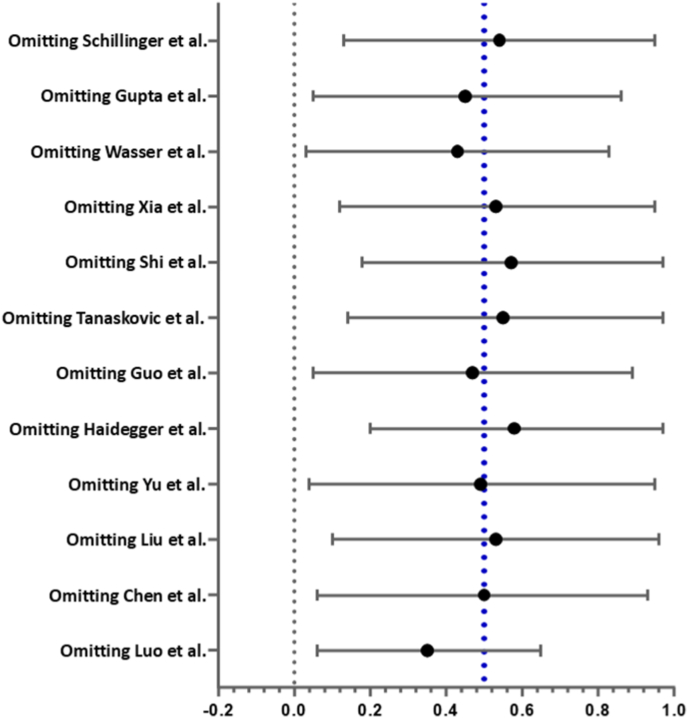
Leave-one-out sensitivity analysis.

The pooled analysis of 12 individual case-control studies demonstrated a significant association between higher baseline CRP levels and the incidence of carotid or IC artery restenosis (SMD = 0.50; 95% CI = 0.11–0.89, p = 0.01) **(**Fig. 2**.**). No evidence of publication bias was observed, either visually using Begg's funnel plots or statistically using Egger's test (p = 0.51) **(**Fig. 3**.**). The derived results remained consistent in our leave-one-out sensitivity analysis (Fig. 4.). A further confounder-based sensitivity analysis which tested for age, gender and disease severity adjustment amongst the included studies was attempted. Age and gender which are perhaps the most important confounders were found to be unmatched amongst restenosis and no-restenosis patient groups in one single study [21], whilst 3 studies [23,24,26] did not report age or gender distributions amongst the two patient groups. Disease severity, on the other hand, was not reported consistently using a standard methodology or not reported at all amongst any of our included studies. Sensitivity analysis after removing the aforementioned 4 studies, still demonstrated a significant association (SMD = 0.56, 95% CI = 0.06–1.07, p = 0.03), and further attested the robustness of our originally derived estimates (Supplementary Figure 1.).

## 4 Discussion

The present study-level meta-analysis of 12 case-control studies reports a statistically significant association between higher pre-procedural CRP levels and the subsequent incidence of carotid and IC restenosis. The obtained effect size was moderate, but robust, as confirmed in our leave-one-out sensitivity analysis. These results align with the prior understanding of the role of CRP in restenosis. The present data suggests that systemic inflammation at the time of index revascularization, as indicated by elevated CRP levels, may serve as a predictor of carotid and intracranial restenosis.

The role of CRP in coronary restenosis has already been extensively studied [12,33,34]. In contrast, only a limited number of studies have focused on exploring risk factors for carotid [35] and intracranial restenosis [36]. This study is the first to summarise the existing evidence using meta-analytic methods and to suggest an association between elevated preprocedural CRP levels and restenosis of carotid and IC lesions. The strengths of our present work include a comprehensive and replicative search strategy, rigorous inclusion/exclusion criteria, independent data extraction by two blinded accessors, as well as a rigorous study-quality assessment strategy, followed by appropriate statistical analysis for extracting pooled effect sizes. Additionally, the robustness of our obtained results was confirmed using leave-one-out sensitivity analyses, as well as stringent assessment of publication bias. Cofounder-based sensitivity analysis further attested our originally obtained pooled results.

The Centers for Disease Control/American Heart Association recommends hs-CRP cut points of <1, 1 to 3, and >3 mg/L into low-, average-, and high-risk categories, respectively [37]. However, in practice, hs-CRP of even >1 mg/L, amongst patients of stable coronary artery disease was shown to be a significant predictor of stroke, independent of baseline characteristics and treatments in a cohort of 3771 patients, evaluated in a randomised trial setting [38]. This suggests compounded risk in patients with high preprocedural CRP levels, since and carotid and IC lesions independently are known to be associated with high incidence of strokes and TIA's [4,6]. Patients with elevated CRP levels and pre-existing atherosclerotic disease, therefore represent the patient cohort that could benefit from aggressive anti-inflammatory treatments. However, while there has been an association between elevations in inflammatory markers and restenosis risk, there is limited evidence suggesting that anti-inflammatory treatments reduce the risk of restenosis; this is a topic worthy of further investigation.

Colchicine, an established anti-inflammatory drug, typically used in treatment of gout, has been extensively tested regarding its efficacy in secondary prevention of cardiovascular disease. Colchicine demonstrated marked reduction in the incidence of a composite endpoint of cardiovascular death, myocardial infarction, stroke, or ischemia-driven revascularization amongst a large cohort of chronic CAD patients in the multicentric, randomised, placebo controlled, Low-Dose Colchicine 2 (LoDoCo2) trial [39]. A biomarker substudy of 278 patients enrolled in LoDoCo2 trial, suggested marked reduction in serum hs-CRP levels amongst patients treated with colchicine compared to those with placebo (median 0.80 versus 1.34 mg/L respectively, difference −0.54 mg/L, 95% CI -0.58 to −0.12 mg/L, p < 0.005), suggesting that benefits in terms of event rate were indeed brought about by colchicine's anti-inflammatory properties [40]. Risk stratification using pre-intervention CRP levels of patients at the time of index intervention was able to identify patients at increased risk. Current Standards of Practice on Carotid Artery Stenting (CIRSE) guidelines of 2024 do not include CRP measurements as a standardized pre-procedural assessment, [41]. Incorporating pre-procedural CRP measurements could identify patients at a higher risk for restenosis. Whether these patients would benefit from intensified follow up or anti-inflammatory medications like colchicine should be investigated in future randomized trials.

### 4.1 Limitations

The present study-level meta-analysis has several limitations. There was high in-group heterogeneity, which was a result of varied study designs, intervention types, and a variety of imaging modalities and cut-offs for defining restenosis, along with variations in follow-up durations amongst the included studies; all of which could have had a bearing on our derived results. Although results of our cofounder-based sensitivity analysis involving factors such as age and gender suggested lack of bias, residual effects on the pooled estimates originating from other important unmatched cofounders such as disease severity etc., however, were not duly addressed because of the lack of methodological uniformity in disease severity assessment or simply lack of relevant published data across the included studies. This can be classified as one of the important limitations of the present analysis. There was also a lack of independent assessment for restenosis, which ideally should be done by blinded assessors to avoid bias. The varied immunoassays used for the measurement of CRP levels across the included studies could have contributed towards heterogeneity and may have had a bearing on the reported results. Most of the included studies had limited sample size and were retrospective case-control or cohort studies. Such studies are traditionally associated with limited statistical power and often report inflated effect sizes due to the ‘small-study effect’.

## 5 Conclusions

The current systematic review and study level meta-analysis of non-randomised studies suggests that there is an association between elevated baseline CRP levels and the subsequent incidence of carotid and intracranial artery restenosis.

## Funding

None.

## Conflict of interest

The authors declare the following financial interests/personal relationships which may be considered as potential competing interests:Prof Robert A. Byrne reports a relationship with Abbott Vascular Inc that includes: funding grants. Prof Robert A. Byrne reports a relationship with Boston Scientific Corporation that includes: funding grants. Prof Robert A. Byrne reports a relationship with Teleflex that includes: funding grants. Prof Robert A. Byrne reports a relationship with Terumo Interventional Systems that includes: funding grants. If there are other authors, they declare that they have no known competing financial interests or personal relationships that could have appeared to influence the work reported in this paper.
