# Supplementary material for: Pre-procedural C-reactive protein levels and carotid or intracranial artery restenosis: A systematic review and meta-analysis
Source: Atheroscler Plus. 2026 Jan 31;63:52–7. doi: 10.1016/j.athplu.2026.01.006 (PMC12907718; doi:10.1016/j.athplu.2026.01.006)
Supplement: Multimedia component 1 [file mmc1.pdf]

Supplementary Table 1. Search strings used for literature search

Database	Search string	Until (Date)	Number of results retrieved
PubMed	((((((((CRP[Title/Abstract]) OR ("C-reactive protein"[Title/Abstract])) OR ("C-Reactive Protein"[MeSH Terms])) OR ("high sensitivity C-reactive protein"[Title/Abstract])) OR (hsCRP[Title/Abstract])) OR (hs-CRP[Title/Abstract])) AND (((((((((angioplasty[Title/Abstract]) OR (stent[Title/Abstract])) OR ("endovascular treatment"[Title/Abstract])) OR ("endoluminal treatment"[Title/Abstract])) OR ("transluminal Treatment"[Title/Abstract])) OR (intervention[Title/Abstract])) OR (bypass[Title/Abstract])) OR (surgery[Title/Abstract])) OR (reconstruction[Title/Abstract])) OR (endarterectomy[Title/Abstract]))) AND ((((((("carotid artery diseases"[MeSh Terms]) OR ("carotid artery disease"[Title/Abstract])) OR ("carotid arter*"[Title/Abstract])) OR (intracranial arterial diseases[MeSh Terms])) OR (intracranial arter*[Title/Abstract])) OR (intracranial[Title/Abstract])) OR (carotid[Title/Abstract]))) AND (((((((restenosis[Title/Abstract]) OR (re-stenosis[Title/Abstract])) OR (revasculari* [Title/Abstract])) OR (re-intervention[Title/Abstract])) OR ("repeat intervention"[Title/Abstract])) OR ("disease progression"[Title/Abstract])) OR ("recurrent stenosis" [Title/Abstract]))) NOT (((((rat[Title/Abstract]) OR (mouse[Title/Abstract])) OR (mice[Title/Abstract])) OR (rabbit[Title/Abstract])) OR ("takayasu arteritis"[Title/Abstract]))	31st Aug 2025	56
EMBASE	(crp:ti,ab OR 'c reactive protein':ti,ab OR 'high sensitivity c reactive protein':ti,ab OR hscrp:ti,ab OR 'hs crp':ti,ab) AND (angioplasty:ti,ab OR stent:ti,ab OR 'endovascular treatment':ti,ab OR 'endoluminal treatment':ti,ab OR 'transluminal treatment':ti,ab OR intervention:ti,ab OR bypass:ti,ab OR surgery:ti,ab OR reconstruction:ti,ab OR endarterectomy:ti,ab) AND ('carotid artery disease':ti,ab OR 'carotid arter*':ti,ab OR 'intracranial arterial disease':ti,ab OR 'intracranial arter*':ti,ab OR intracranial:ti,ab OR carotid:ti,ab) AND (restenosis:ti,ab OR 're stenosis':ti,ab OR revasculari*:ti,ab OR 're intervention':ti,ab OR 'repeat intervention':ti,ab OR 'disease progression':ti,ab OR 'recurrent stenosis':ti,ab) NOT (rat:ti,ab OR mouse:ti,ab OR mice:ti,ab OR rabbit:ti,ab OR 'takayasu arteritis':ti,ab OR 'giant cell arteritis':ti,ab OR 'temporal arteritis':ti,ab OR 'coronary artery':ti,ab OR 'coronary artery disease':ti,ab OR 'coronary arteries':ti,ab OR 'coronary arter*':ti,ab OR coronary:ti,ab OR 'myocardial infarction':ti,ab OR	31st Aug 2025	55

	'myocardial infarct*':ti,ab OR 'heart attack':ti,ab OR 'acute coronary syndrome':ti,ab OR acs:ti,ab OR 'peripheral artery':ti,ab OR pad:ti,ab OR femoral:ti,ab OR popliteal:ti,ab OR 'heart surgery':ti,ab OR 'cardiac surgery':ti,ab OR cabg:ti,ab)		
SCOPUS	TITLE-ABS-KEY (CRP OR "c reactive protein" OR "high sensitivity c reactive protein" OR hsCRP OR hs-CRP) AND TITLE-ABS-KEY (angioplasty OR stent OR "endovascular treatment" OR "endoluminal treatment" OR "transluminal treatment" OR intervention OR bypass OR surgery OR reconstruction OR endarterectomy) AND TITLE-ABS-KEY ("carotid artery disease" OR "carotid arter*" OR "intracranial arterial disease" OR "intracranial arter*" OR intracranial OR carotid) AND TITLE-ABS-KEY (restenosis OR re-stenosis OR revasculari* OR re-intervention OR "repeat intervention" OR "disease progression" OR "recurrent stenosis") AND NOT TITLE-ABS-KEY (rat OR mouse OR mice OR rabbit OR "takayasu arteritis" OR "giant cell arteritis" OR "temporal arteritis" OR "coronary artery" OR "coronary artery disease" OR "coronary arteries" OR "coronary arter*" OR coronary OR "myocardial infarction" OR "myocardial infarct*" OR "heart attack" OR "acute coronary syndrome" OR ACS OR "peripheral artery" OR PAD OR femoral OR popliteal OR "heart surgery" OR "cardiac surgery" OR CABG)	31st Aug 2025	100
MEDLINE	(((CRP or c reactive protein).mp. or exp C-Reactive Protein/ or high sensitivity c reactive protein.mp. or hsCRP.mp. or hs-CRP.mp.) and (angioplasty or stent or endovascular treatment or endoluminal treatment or transluminal treatment or intervention or bypass or surgery or reconstruction or endarterectomy).mp. and (exp Carotid Artery Diseases/ or carotid artery disease.mp. or carotid arter*.mp. or exp Intracranial Arterial Diseases/ or intracranial arter*.mp. or intracranial.mp. or carotid.mp.) and (restenosis or re-stenosis or revasculari* or re-intervention or "repeat intervention" or "disease progression" or "recurrent stenosis").mp.) not ((rat or mouse or mice or rabbit or takayasu arteritis).mp. or exp Coronary Artery Disease/ or coronary artery.mp. or coronary arter*.mp. or exp Myocardial Infarction/ or myocardial infarction.mp. or myocardial infarct*.mp. or heart attack.mp. or acute coronary syndrome.mp. or ACS.mp. or peripheral artery.mp. or PAD.mp. or femoral.mp. or popliteal.mp. or heart surgery.mp. or cardiac surgery.mp. or CABG.mp. or exp Giant Cell Arteritis/ or giant cell arteritis.mp. or temporal arteritis.mp.)	31st Aug 2025	48
Web of Science	(CRP OR "c reactive protein" OR "high sensitivity c reactive protein" OR hsCRP OR hs-CRP) (Topic)	31st Aug 2025	38

	AND<br>(angioplasty OR stent OR "endovascular treatment" OR "endoluminal treatment" OR "transluminal treatment" OR intervention OR bypass OR surgery OR reconstruction OR endarterectomy) (Topic)<br>AND<br>("carotid artery disease" OR "carotid arter*" OR "intracranial arterial disease" OR "intracranial arter*" OR intracranial OR carotid) (Topic)<br>AND<br>(restenosis OR re-stenosis OR revasculari* OR re-intervention OR "repeat intervention" OR "disease progression" OR "recurrent stenosis") (Topic)<br>NOT<br>(rat OR mouse OR mice OR rabbit OR "takayasu arteritis" OR "giant cell arteritis" OR "temporal arteritis" OR "coronary artery" OR "coronary artery disease" OR "coronary arteries" OR "coronary arter*" OR coronary OR "myocardial infarction" OR "myocardial infarct*" OR "heart attack" OR "acute coronary syndrome" OR ACS OR "peripheral artery" OR PAD OR femoral OR popliteal OR "heart surgery" OR "cardiac surgery" OR CABG)		
