# Supplementary material for: Pre-procedural C-reactive protein levels and carotid or intracranial artery restenosis: A systematic review and meta-analysis
Source: Atheroscler Plus. 2026 Jan 31;63:52–7. doi: 10.1016/j.athplu.2026.01.006 (PMC12907718; doi:10.1016/j.athplu.2026.01.006)
Supplement: Multimedia component 2 [file mmc2.pdf]

<u>Supplementary Table 2.</u> List of excluded studies

S.no.	Author	Year	Citation	Reason for exclusion
1	Schillinger *et al.*	2003	Circulation. 2003 Nov 11;108(19):2323-8.	Other endpoints (not restenosis)
2	Schillinger *et al.*	2004	J Endovasc Ther. 2004 Jun;11(3):229-39.	Raw C-reactive protein data relevant for analysis not published
3.	Lusic *et al.*	2006	Vasa. 2006 Nov;35(4):221-5.	Full text not accessible online. No response from the lead author even after three email requests for full text.
4.	Széplaki *et al.*	2006	Thromb Haemost. 2006 Oct;96(4):529-34.	Not relevant to current analysis
5.	Arthurs *et al.*	2008	J Vasc Surg. 2008 Apr;47(4):744-50; discussion 751.	Raw C-reactive protein data relevant for analysis not published
6.	Fittipaldi *et al.*	2016	J Cardiovasc Surg (Torino). 2016 Dec;57(6):861-871.	Histological study
7.	Wrotniak *et al.*	2016	J Vasc Surg. 2016 Sep;64(3):684-91.	Raw data not published (also, subclavian artery disease)
8.	Stone *et al.*	2017	Ann Vasc Surg. 2017 Jan;38:144-150.	Other endpoints (not restenosis)
9.	Foroughinia *et al.*	2019	Caspian J Intern Med. 2019 Fall;10(4):388-395.	C-reactive protein sample taken after index procedure
10.	Biscetti *et al.*	2021	Cardiovasc Diabetol. 2021 May 27;20(1):114.	No follow-up after index procedure
11.	Yu *et al.*	2023	Stroke Vasc Neurol. 2024 Aug 27;9(4):407-417.	Raw C-reactive protein data relevant for analysis not published
12.	Durmuş *et al.*	2024	Angiology. 2024 Aug 19:33197241273331.	Full text not accessible online. No response from the lead author even after three email requests for full text.
13.	Wu et al.	2025	Neurologist. 2025 Jun 11.	Not relevant to current analysis
