# Supplementary figures and images for: Pre-procedural C-reactive protein levels and carotid or intracranial artery restenosis: A systematic review and meta-analysis
Source: Atheroscler Plus. 2026 Jan 31;63:52–7. doi: 10.1016/j.athplu.2026.01.006 (PMC12907718; doi:10.1016/j.athplu.2026.01.006)

**Supplementary Figure 1. Cofounder-based sensitivity analysis**

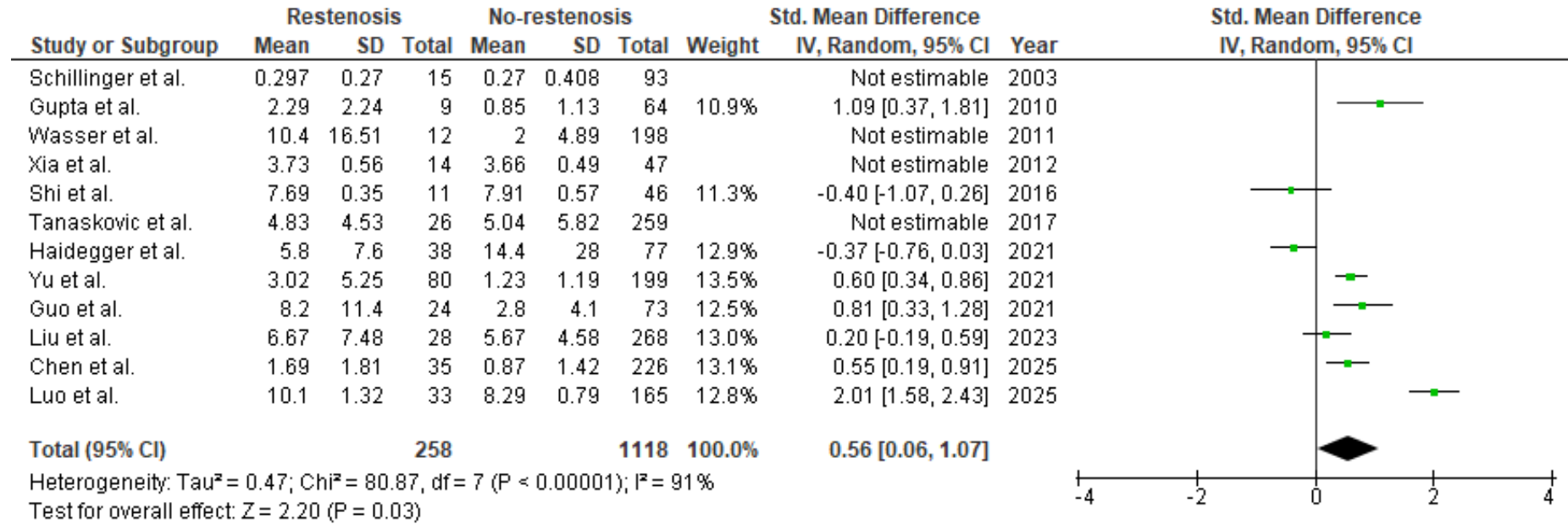

Supplement: Multimedia component 3 [file mmc3.pdf]
